# Factors affecting occupational burnout in medical staff: a path analysis based on the job demands-resources perspective

**DOI:** 10.3389/fpsyt.2024.1490171

**Published:** 2024-10-21

**Authors:** Zuolin Wei, Bocheng Xia, Lingli Jiang, Huaiyi Zhu, Lingyan Li, Lin Wang, Jun Zhao, Ruoxin Fan, Peng Wang, Mingjin Huang

**Affiliations:** ^1^ School of Clinical Medicine, North Sichuan Medical College, Nanchong, China; ^2^ The Third Hospital of Mianyang, Sichuan Mental Health Center, Mianyang, Sichuan, China

**Keywords:** burnout, job demands-resources, medical staff, workplace support, COVID-19

## Abstract

**Objective:**

To assess occupational burnout conditions and work-related factors among frontline medical staff during the COVID-19 pandemic and analyse the relationships among these factors utilizing the job demands–resources (JD-R) model as a theoretical framework.

**Methods:**

An online survey was distributed to medical staff in one city via convenience sampling during 12/29/2022–1/10/2023. Path analysis was utilized to explore the relationship between work-related factors and occupational burnout among frontline medical staff during the COVID-19 outbreak.

**Results:**

Among 474 respondents, 455 frontline medical staff (female=79.56%) were included in the final analysis. Medical staff aged <35 exhibited higher levels of occupational burnout than did older staff. Depression/anxiety and workload were positively correlated with occupational burnout and negatively correlated with self-compassion, workplace health/safety, and workplace support. Path analysis indicated the direct effects of workplace support, depression/anxiety, workplace health/safety, self-compassion, and workload on occupational burnout. There were also partial mediating effects of workplace support, depression/anxiety, workplace health/safety, and self-compassion on occupational burnout. The model demonstrated good fit.

**Conclusion:**

Workplace support, a crucial job resource, can improve occupational burnout among frontline medical staff in various ways. Reducing anxiety, depression, and workload and improving workplace support, health/safety, and self-compassion are practical and effective measures for mitigating occupational burnout.

## 1 Introduction

High-demand work environments can induce a persistent negative emotional state known as burnout (1). The likelihood of occupational burnout increases when work demands are excessive or when work resources are diminished (2). Burnout encompasses three dimensions—emotional exhaustion, depersonalization, and reduced personal accomplishment (3)—with varying manifestations across different professions. For medical staff, emotional exhaustion manifests as physical and mental fatigue at the end of a workday and a strong need for rest, along with an inability to provide emotional support to patients; depersonalization is reflected in treating patients as objects rather than human beings, leading to a callous attitude towards patients; and reduced personal accomplishment is characterized by feeling ineffective in helping patients and lacking a sense of value in patient care or professional achievements (4). While occupational burnout can affect all industries, it is particularly pronounced in the medical field. Prior to the COVID-19 outbreak, a meta-analysis covering 45 countries revealed that the global burnout prevalence among nurses was 11.23% (5). During the COVID-19 pandemic, medical staff faced high workloads, shortages of supplies, and infection concerns, all of which severely challenged their mental health (6). Studies indicate that the occupational burnout prevalence among medical staff during the pandemic reached 54.60% (95% CI: 46.70%-62.20%) (7). Occupational burnout in medical staff is associated with psychological issues, such as anxiety and depression, and can lead to increased absenteeism, higher turnover rates, and a decline in the quality of medical services and patient satisfaction (8–11). The impact of occupational burnout on medical staff is extensive, affecting staff, patients, organizations, and society at large, making in-depth research on this issue crucial.

We utilized the job demands–resources (JD-R) model to explore the factors influencing burnout (12). This model identifies two categories of risk factors related to occupational burnout: job demands and job resources. ‘Job demands’ refers to the cognitive and emotional efforts involved in work, typically associated with psychological costs. Alternatively, ‘job resources’ encompasses material, sociopsychological, and organizational support, aiding in achieving work objectives and personal development and alleviating work stress (13, 14). Long-term exposure to an imbalance between job demands and resources may lead to burnout. Difficulties encountered by medical staff in clinical work, such as increased workload, organizational conflict, lack of skills, and insufficient social support, which subsequently increase the risk of anxiety, depression, and burnout, are considered job demands (15, 16). Conversely, providing medical staff with a healthy and safe work environment, good teamwork, and leadership recognition are viewed as job resources (17, 18). Self-compassion, which enables individuals to better cope with work stress and helps alleviate symptoms of depression, anxiety, and burnout, can also be considered a job resource (19–21).

**Figure 1:**
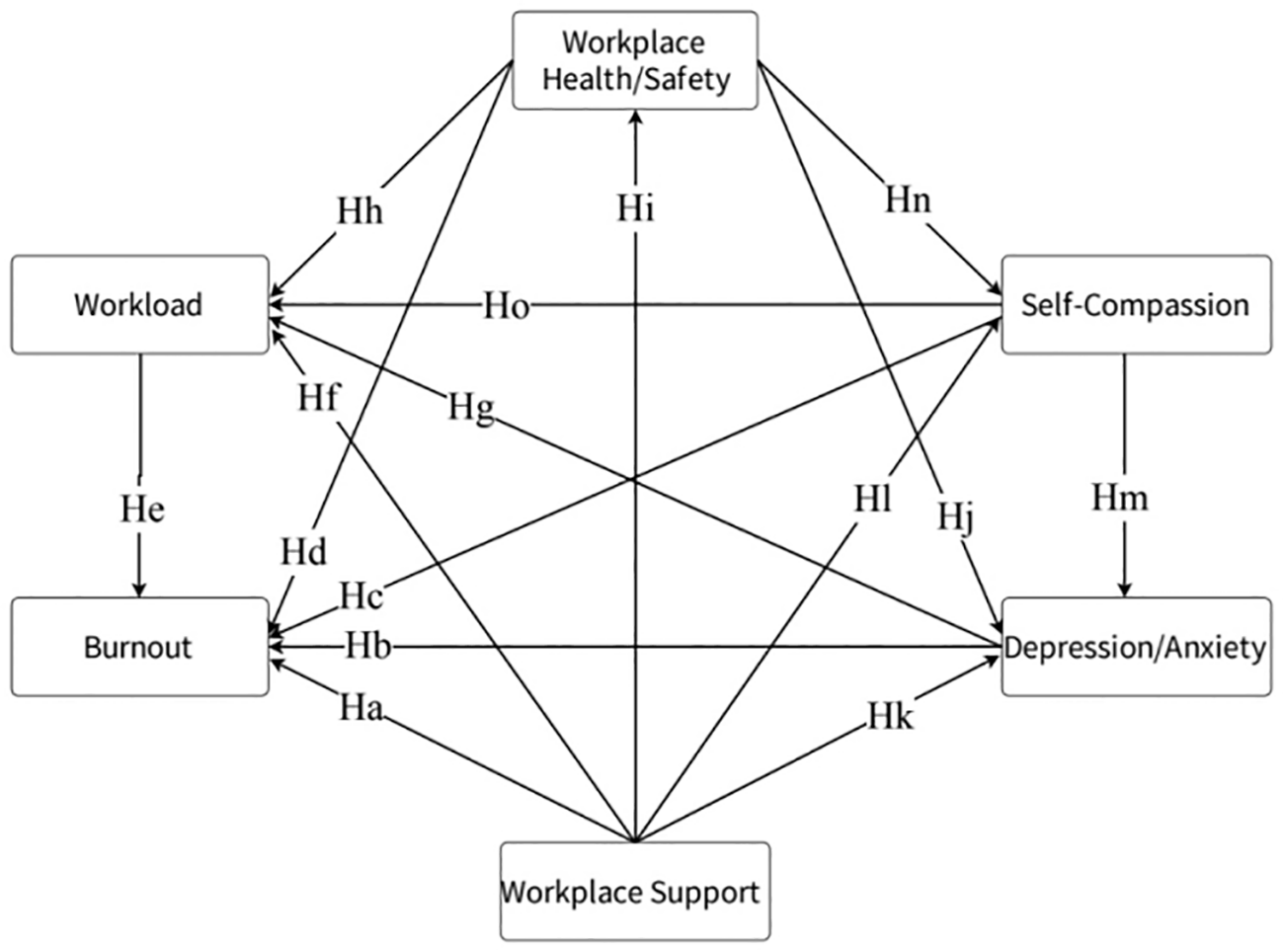
Hypothetical model. The hypothesized relationships between the variables are illustrated in the figure (e.g., Ha indicates that workplace support affects occupational burnout).

During this phase, the psychological health risks of Chinese medical staff have reached a rare peak due to the COVID-19 pandemic. Their unique working model and significant occupational burnout issues present a unique opportunity to explore the factors influencing occupational burnout. Integrating previous research and the JD-R theory, we believe that the job demands of medical staff (e.g., depression/anxiety, workload) exacerbate the risk of occupational burnout. On the other hand, job resources (e.g., workplace support, health/safety, self-compassion) are crucial for alleviating occupational burnout and maintaining mental health. However, there is currently limited research revealing how these factors influence burnout through comprehensive models. Considering previous theoretical studies and the hypothesized relationships between job demands, job resources, and occupational burnout, we formulated the following hypotheses (**Figure 1**):

### 1.1 Hypotheses with workplace support as the starting point of the arrow

Workplace support is negatively correlated with occupational burnout (Hypothesis a, (Ha)), workload (Hf), and depression/anxiety (Hk) and is positively correlated with workplace health/safety (Hi) and self-compassion (Hl).

### 1.2 Hypotheses with depression/anxiety as the starting point of the arrow

Depression/anxiety are positively correlated with occupational burnout (Hb) and workload (Hg).

### 1.3 Hypotheses with self-compassion as the starting point of the arrow

Self-compassion is negatively correlated with occupational burnout (Hc), workload (Ho), and depression/anxiety (Hm).

### 1.4 Hypotheses with workplace health/safety as the starting point of the arrow

Workplace health/safety is negatively correlated with occupational burnout (Hd), workload (Hh), and depression/anxiety (Hj) and positively correlated with self-compassion (Hn).

### 1.5 Hypotheses with workload as the starting point of the arrow

Workload is positively correlated with occupational burnout (He).

## 2 Materials and methods

### 2.1 Study design and participants

We employed a convenience sampling method, utilizing both an online questionnaire collection tool, Questionnaire Star (https://www.wjx.cn, Changsha, China), and offline paper questionnaires. During January 2023, amidst the COVID-19 pandemic, these questionnaires were distributed to medical staff in healthcare institutions in Mianyang city, and all active medical personnel were eligible to participate in the survey. All respondents voluntarily and anonymously completed the online questionnaire. Each IP address was allowed to submit the questionnaire only once. All participants received full disclosure of information and provided informed consent. The inclusion criteria were as follows: (1) aged 18–65 years and (2) actively engaged in clinical work during this period. We excluded non-frontline staff (those who selected options such as “medical technician/medical professional (laboratory, technical department, X-ray, pharmacy)”, “management”, “administrative logistics” or “other” in the “nature of work” section of the questionnaire).

### 2.2 Assessment tools

#### 2.2.1 Demographic questionnaire

The demographic and occupational characteristics surveyed included sex, age, years of work experience, nature of work, and likelihood of infection.

#### 2.2.2 Oldenburg burnout inventory

The degree of occupational burnout was measured using the OLBI, developed in 2003 by German psychologist Demerouti (22). The OLBI encompasses two dimensions: exhaustion and disengagement. Exhaustion refers to a state of overstrain at work, a strong need for rest, and physical fatigue, while disengagement is characterized by distancing oneself from work tasks and content, along with a negative attitude and behaviour towards work. The OLBI consists of 16 items, each assessed using a 4-point Likert scale. We extracted items 4 and 14 to measure the exhaustion dimension (e.g., “After work, I need more time than before to relax and feel better. “) and items 3, 7, and 11 to measure the disengagement dimension (e.g., “When I talk about my work, it is mostly negative content.”). Items 3, 4, and 11 are reverse-scored. A higher total score indicates a greater degree of burnout. In this sample, Cronbach’s alpha=0.56.

#### 2.2.3 PHQ-2 and GAD-2

Depression/Anxiety. The Composite Depression/Anxiety Screening Scale includes two core anxiety items (Generalized Anxiety Disorder-2, GAD-2) and two core depression items (Patient Health Questionnaire-2, PHQ-2) and is currently the shortest effective composite measure for assessing depression and anxiety disorders (23). Responses are rated on a 4-point scale (0=“Not at all” to 3=“Nearly every day”). A higher total score indicates a greater likelihood of depression or anxiety (Cronbach’s alpha=0.91).

#### 2.2.4 Self-compassion scale

The widely used SCS, initially developed by Neff (24), measures an individual’s self-compassionate response to stressful experiences through six components: self-kindness, overidentification, common humanity, self-judgement, mindfulness, and isolation. State self-compassion is derived from the SCS by asking participants to focus on their current pain or difficulty and choose options that reflect their situation (e.g., “I give myself the care and tenderness I need.”) (19). Responses are rated on a 5-point scale (1=“Not at all true” to 5=“Very true”). A higher total score indicates a greater level of self-compassion (Cronbach’s alpha=0.65).

#### 2.2.5 Support at the workplace

Five items were used to assess the support situation in the workplace (e.g., “My team is currently cooperative and supportive of each other.”) (17). All items are rated on a 4-point scale (1=“Strongly disagree” to 4=“Strongly agree”). A higher total score indicates greater support provided in the workplace (Cronbach’s alpha=0.85).

#### 2.2.6 Health and safety at the workplace

Two items were used to measure subjective perceptions of health and safety in the workplace (17). One item covered the availability of personal protective equipment (such as masks, gloves, sanitizers, etc.), and the other covered participants’ “confidence in staying healthy at work.” Both items are rated on a 5-point scale (“1=Strongly agree” to “5=Strongly disagree”) and reverse-scored. A higher total score indicates a stronger sense of health/safety (Cronbach’s alpha=0.50).

#### 2.2.7 Workload

Three items were used to assess current work conditions from the perspectives of workload, sufficient staffing, and adequate rest after work (e.g., “Compared to before the COVID-19 pandemic, my workload has increased.”). All items are rated on a 5-point scale (0=“Strongly disagree” to 4=“Strongly agree”). Items 2 and 3 are reverse-scored, with a higher total score indicating a greater workload (Cronbach’s alpha=0.68).

### 2.3 Statistical analysis

The data were statistically analysed using IBM SPSS 27.0 and AMOS 26.0, with descriptive analysis employed to display the demographic indicators. For analysis of between-group differences, one-way ANOVA was utilized. Pearson correlation analysis was conducted to explore the relationships between depression/anxiety, workplace support, workplace health/safety, self-compassion, work conditions, and occupational burnout. Path analysis was employed to assess the direct and mediating effects of potential predictor variables on outcome variables, with the maximum likelihood method used to test the impact of independent variables on burnout.

## 3 Results

### 3.1 Participant characteristics

In total, 474 complete datasets were collected online and offline. After excluding 19 datasets from non-frontline staff, 455 datasets from frontline medical personnel were included in the analysis (response rate=95.99%). Among the 455 valid questionnaires collected, the majority were from female respondents (79.56%), participants <35 years old (71.43%), those with >6 years of work experience (50.55%), and nurses (59.78%); 374 (82.20%) had been infected with COVID-19 during the survey period, and none selected the 0-point option (“highly unlikely”) (**Table 1**). Additionally, age was the only demographic factor significantly associated with occupational burnout. Medical staff <35 years old exhibited greater levels of burnout than did those in other age groups (p<0.01).

**Table 1:** Demographic information of the study participants and its relationship with occupational burnout.

Variables	Items	Frequency (N=455)	(%)	Burnout	
				*F*	*P*
Sex				3.60	0.06
	Male	93	20.44		
	Female	362	79.56		
Age				7.71	0.01
	<35	325	71.43		
	>35	130	28.57		
Work seniority				1.14	0.32
	<3 years	149	32.75		
	3-6 years	76	16.70		
	>6 years	230	50.55		
Job category				1.64	0.20
	Doctor	124	27.25		
	Nurse	272	59.78		
	Other	59	12.97		
Infection potential				1.83	0.12
	rather unlikely	3	0.66		
	uncertain	23	5.05		
	rather likely	20	4.40		
	highly likely	35	7.69		
	infected	374	82.20		

### 3.2 Correlation relationships

Pearson correlation analysis revealed that anxiety/depression (r=0.54, p<0.01) and workload (r=0.49, p<0.01) were significantly positively correlated with burnout, while self-compassion (r=-0.41, p<0.01), workplace health/safety (r=0.40, p<0.01), and workplace support (r=-0.45, p<0.01) were significantly negatively correlated with burnout (**Table 2**).

**Table 2:** Pearson correlations among all study variables (N=457).

Variables	1	2	3	4	5	6
1. Burnout	1.00					
2. Workplace Support	-0.45**	1.00				
3. Depression/Anxiety	0.54**	-0.28**	1.00			
4. Self-Compassion	-0.41**	0.27**	-0.43**	1.00		
5. Workplace Health/Safety	-0.40**	0.34**	-0.36**	0.20**	1.00	
6. Workload	0.49**	-0.41**	0.42**	-0.23**	-0.39**	1.00

** p<0.01.

### 3.3 Path analysis

The model fitting results indicate that the fit indices, such as chi-square/df=3.358, are within the recommended range of 1-5 (25). The root mean square error of approximation (RMSEA) is 0.07, which is less than the critical value of 0.08 suggested by Steiger (26). The goodness-of-fit indices, such as the NFI, IFI, TLI, CFI, and GFI, all exceeded the recommended value of 0.90 (NFI=0.990, IFI=0.993, TFI=0.997, CFI=0.993, GFI=0.995) (27), suggesting that the model had a good fit.

**Figure 2:**
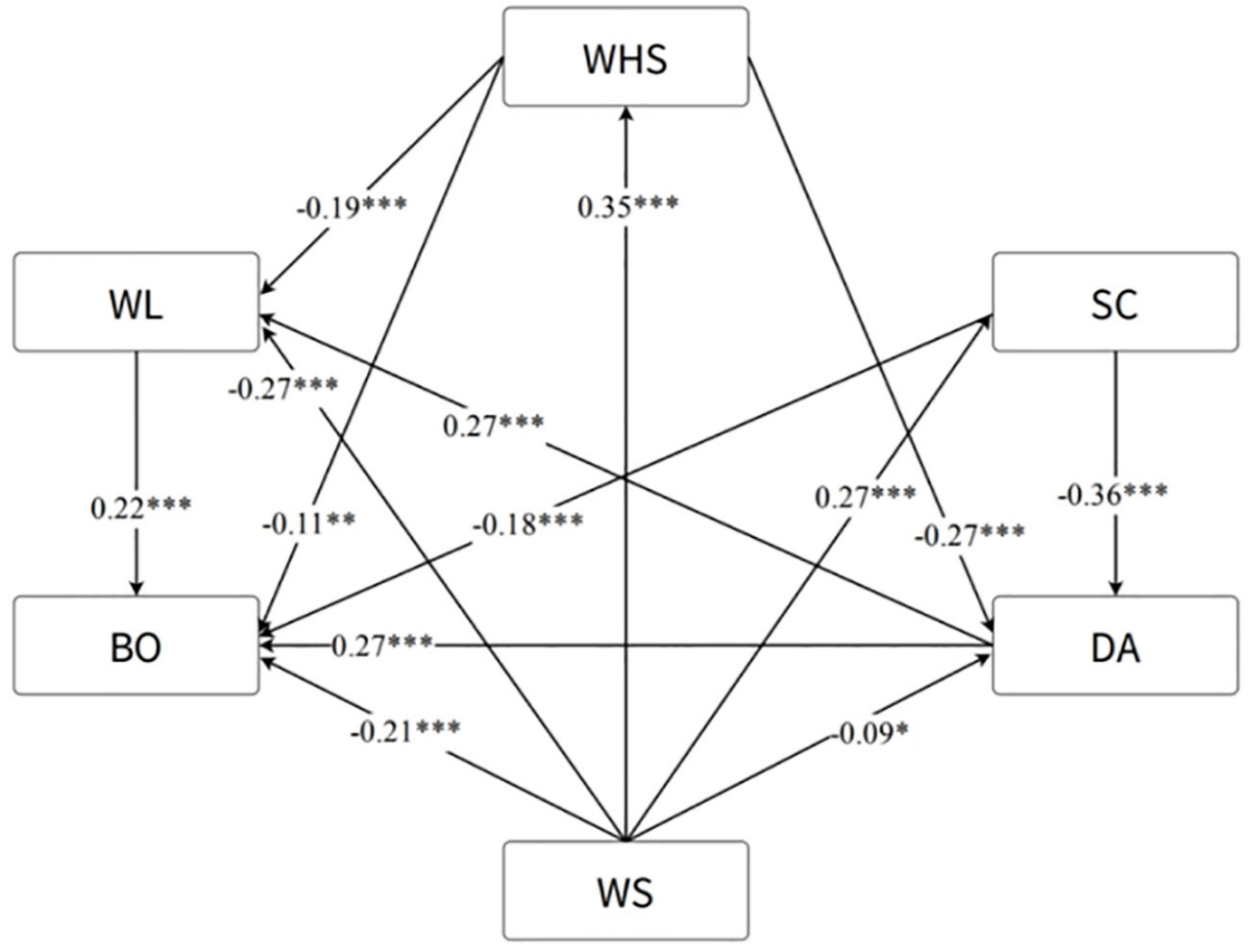
Final path analysis results among the various study variables. WL, workload; BO, burnout; WHS, workplace health/safety; WS, workplace support; SC, self-compassion; DA, depression/anxiety. *p<0.05, ** p<0.01, *** p<0.001.

The standardized regression coefficients and significance levels of the hypothesized model are shown in **Figure 2**, and **Table 3** presents the specific direct, indirect, and total effect results. Lower workplace support (β=-0.21, P<0.001), self-compassion (β=-0.18, P<0.001), and workplace health/safety (β=-0.11, P<0.01) were significantly associated with greater occupational burnout, supporting hypotheses Ha, Hc, and Hd (**Figure 2**). Higher levels of depression/anxiety (β=0.27, P<0.001) and workload (β=0.22, P<0.001) were significantly related to greater occupational burnout, supporting hypotheses Hb and He. Lower workplace support was significantly associated with greater depression/anxiety (β=-0.09, P<0.05) and workload (β=-0.27, P<0.001), supporting hypotheses Hk and Hf, and lower workplace health/safety (β=0.35, P<0.001) and self-compassion (β=0.27, P<0.001), supporting hypotheses Hi and Hl. Higher workplace health/safety was significantly associated with lower workload (β=-0.19, P<0.001) and depression/anxiety (β=-0.265, P<0.001) but not self-compassion (P>0.05), supporting Hh and Hj but not Hn. Considering that greater depression/anxiety (β=0.27, P<0.001) was related to a greater workload, supporting Hg, workplace health/safety partially mediate the effects of depression/anxiety on workload. Lower self-compassion was significantly related to greater depression/anxiety but not workload (P>0.05), supporting Hm but not Ho; therefore, depression/anxiety fully mediated the relationship between self-compassion and workload. Notably, workplace support had the greatest total and indirect effects on occupational burnout, while depression/anxiety had the greatest direct effects on occupational burnout (**Table 3**).

**Table 3:** Direct, indirect, and total effects results for significant pathways.

Hypothesis	Relationships	Total	Direct effects	Indirect effects
Ha	WS→BO	-0.46	-0.21	-0.25
Hb	DA→BO	0.33	0.27	0.06
Hc	SC→BO	-0.30	-0.18	-0.12
Hd	WHS→BO	-0.24	-0.11	-0.13
He	WL→BO	0.22	0.22	–
Hf	WS→WL	-0.42	-0.27	-0.15
Hg	DA→WL	0.27	0.27	–
Hh	WHS→WL	-0.27	-0.19	-0.08
Hi	WS→WHS	0.35	0.35	–
Hj	WHS→DA	-0.27	-0.27	–
Hk	WS→DA	-0.28	-0.09	-0.19
Hl	WS→SC	0.27	0.27	–
Hm	SC→DA	-0.36	-0.36	–
Hm*Hg	SC→→WL	-0.10	–	-0.10

WL, workload; BO, burnout; WHS, workplace health/safety; WS, workplace support; SC, self-compassion; DA, depression/anxiety. Hypothesized paths Hn and Ho were nonsignificant, so they were omitted.

## 4 Discussion

We utilized a comprehensive model to assess the relationships of workplace support, workplace health/safety, self-compassion, anxiety/depression, and workload with occupational burnout while exploring their mediating effects on the workplace support and occupational burnout association.

The COVID-19 crisis has brought greater challenges to an already heavily burdened healthcare system, with different groups exhibiting varying sensitivities to occupational burnout. We found that medical personnel aged <35 experienced more severe occupational burnout than those in other age groups, suggesting that younger doctors are more prone to burnout than their older counterparts. This finding is consistent with those of Novilla et al. (28), who reported that longer professional practice is correlated with lower levels of burnout, anxiety, and depression. This may be because younger medical staff generally lack experience and skills, increasing their susceptibility to uncertainty and pressure in complex, high-stress medical environments.

Numerous studies have preliminarily shown a close correlation between increased work demands and higher levels of burnout (29, 30). A high number of patients, extended working hours, staff shortages, and inadequate personal protective equipment and resources increase the susceptibility of medical staff to burnout, anxiety, and depression (28, 31). We revealed similar results: depression, anxiety, and workload significantly increased burnout, supporting the hypothetical paths of Hb and He. Additionally, research indicates that anxiety, depression, and stress not only lead to occupational burnout but also affect the work performance of medical staff, reducing the quality of healthcare (11). When the workplace fails to ensure health and safety, medical staff often experience anxiety and depression, which in turn affects their work performance (27) This leads to a decrease in work efficiency and an increase in workload, ultimately resulting in occupational burnout. This prompts us to accept the hypothesis paths Hd, Hg, Hh, and Hj, building on the foundation established earlier.

Self-compassion is a multidimensional structure involving inwardly directed compassion, that is, kindly accepting one’s own failures and shortcomings (24, 32). We showed that self-compassion has a significant negative effect on burnout and anxiety/depression, supporting hypothesis paths Hc and Hm. This is consistent with previous studies showing that nurses with self-compassion who confront their shortcomings with kindness, experience less burnout and compassion fatigue (33). People with strong self-compassion skills are better at discarding negative thoughts, accepting their own and others’ shortcomings, and effectively managing emotions under stress (34). Additionally, individuals with high levels of self-compassion are more effective at managing occupational stress and challenges in therapeutic work, reducing symptoms of anxiety and depression (35, 36). Therefore, self-compassion is an important protective factor in preventing occupational hazards (37). Self-compassion is a skill that can be acquired through learning and practice, with a key aspect being the adoption of a kind and nonjudgmental attitude towards suffering (38, 39). Compassion-focused therapy (CFT) was originally developed for clinical populations experiencing high levels of shame and self-criticism (40), and it has been effective in enhancing self-compassion and treating symptoms such as eating disorders and social anxiety (41). Mindful self-compassion (MSC) is suitable for nonclinical populations (42), helping to increase self-compassion and life satisfaction while reducing depression/anxiety (43) and surpassing cognitive behavioural therapy (CBT) in certain respects (44). Additionally, the Self-Compassion Training Course for Health Communities (SCHC), designed specifically for healthcare professionals, aids in enhancing self-compassion and focus, reducing stress and burnout. This course does not rely on meditation, making it more practical for implementation during workdays (38, 45).

Many studies indicate that workplace support may play a particularly important protective role for medical staff in pandemic environments (46, 47). A qualitative study during the pandemic showed that medical staff who received workplace support had a significantly lower risk of generalized anxiety disorder, clinical insomnia, severe depression, emotional exhaustion, depersonalization, and poor mental health than their unsupported peers. Furthermore, over time, an increase in workplace support is associated with increased well-being and decreased anxiety and depression scores (48). We also found that workplace support is a key factor in reducing occupational burnout and depression/anxiety, supporting hypothesis paths Ha and Hk. Additionally, in previous research (17), we observed that workplace support can reduce occupational burnout at different time points. Creating a work environment full of support and trust is a key resource for enhancing work efficacy. The establishment of trust within and between teams, as well as guidance from superiors and the hospital, is crucial for creating a healthy work environment, leading us to accept hypothesis path Hi, i.e., workplace support can enhance workplace health/safety. Similar to our hypothesis path Hf, a study specifically targeting nurses found that those perceiving that they were part of a good team exhibited lower levels of burnout (18), even with increased workload. Studies show that higher levels of organizational support are associated with higher levels of self-compassion, and an increase in organizational support seems to elevate the self-compassion levels of medical staff (49). The results of hypothesis path Hl affirm this research, suggesting that senior recognition and junior trust, improving teamwork and cross-team communication quality, and providing more comprehensive information might be important measures for enhancing medical staff self-compassion in workplaces.

## 5 Limitations

This study has several limitations. First, a convenience sampling method was employed, which might have affected the representativeness of the sample. Since the sample was not selected randomly, the results may not fully reflect the overall characteristics of the target population. Second, the cross-sectional study design, with data collected at a single time point, prevents the establishment of causal relationships. Additionally, despite efforts to control for major variables, the possibility of residual confounding remains, meaning that other factors not considered could affect the study outcomes. Finally, as this study is limited to a specific region (Mianyang), the generalizability of its conclusions may be limited. Based on these limitations, future research should expand the sample size and employ more rigorous study designs to enhance the generalizability and reliability of the findings.

## 6 Conclusion

The results of this study confirm that factors related to job demands, such as workload and anxiety-depression, exacerbate the negative impact of occupational burnout. In contrast, factors related to job resources, including workplace support, health/safety, and self-compassion, play a significant protective role in alleviating work stress, particularly occupational burnout. Therefore, managers should prioritize the mental health of healthcare workers during major public health events and take measures to enhance job-related resources to reduce occupational burnout.

## Data Availability

The original contributions presented in the study are included in the article/supplementary material. Further inquiries can be directed to the corresponding author.
